# The Characteristics of Emotional Response of Post-traumatic Stress Disorder and Post-traumatic Growth among Chinese Adults Exposed to an Explosion Incident

**DOI:** 10.3389/fpubh.2017.00003

**Published:** 2017-02-08

**Authors:** Chuguang Wei, Jin Han, Yuqing Zhang, Walter Hannak, Zhengkui Liu

**Affiliations:** ^1^Key Laboratory of Mental Health, Institute of Psychology, Chinese Academy of Sciences, Beijing, China; ^2^University of Chinese Academy of Sciences, Beijing, China; ^3^The Core Facility of Institute of Psychology, Chinese Academy of Sciences, Beijing, China; ^4^China Meteorological Administration Training Centre, Beijing, China

**Keywords:** trauma, post-traumatic stress disorder, post-traumatic growth, emotional response, disaster

## Abstract

**Purpose:**

Post-traumatic stress disorder (PTSD) and post-traumatic growth (PTG) are two different outcomes that may occur after experiencing traumatic events. Meanwhile, the traumatic exposure level and emotion response played an important role in the process. The present study first evaluated the relationship between PTSD, PTG, and traumatic exposure level and then compared the characteristics of emotional response through response time of the affective priming paradigm.

**Methods:**

For the purpose of evaluating the relationship between PTSD, PTG, and trauma exposure level, a sample of 2,395 participants completed measures of posttraumatic stress disorder Checklist-Civilian Version (PCL-C), Post-traumatic Growth Inventory (PTGI) and a trauma exposure-related survey, and Pearson’s correlation analysis for the scales were conducted. In order to compare the characteristics of emotional response between PTSD and PTG, we randomly selected 90 participants and divided them into groups of PTSD, PTG, and control according the scores of PCL-C and PTGI, then the 90 participants were asked to do the affective priming task and the response time was recorded, at last analysis of variance was employed to analyze the data.

**Results:**

The results indicated that PTSD was not correlated with PTG. It was positively correlated with the traumatic exposure level, but PTG was not observed in this phenomenon. Finally, the data of response time showed that PTSD required more time to do the priming task and PTG demonstrated no difference compared to the control group.

**Conclusion:**

Combined with previous research findings, the relationship between PTSD and PTG may depend on the type and severity of the trauma, the exposure level, and other such parameters. In terms of positive outcome of trauma PTG displayed no changes of emotional performance from the perspective of behavior. The preliminary results suggested that PTG was more related to a self-reported or self-experienced state.

## Introduction

Life-threatening illnesses and events such as earthquakes, motor vehicle accidents, or terror incidents may cause post-traumatic stress disorders (PTSD) and post-traumatic growth (PTG) (1–5). PTSD refers to a series of symptoms that individuals manifested when dealing with sudden, threatening, or disastrous events. Since the development of a new chapter in DSM-V on trauma- and stress-related disorders, much more attention has been paid to the emotional symptoms that accompany PTSD. The proposed four distinct diagnostic clusters are described as re-experiencing, avoidance, negative cognitions and mood, and arousal (6). Over the past decades, a great deal of research work has focused on PTSD, including the theory, differential diagnosis, the neural mechanisms, and other factors.

Meanwhile, the research on the positive changes following trauma have received less attention. PTG refers to the experience of positive change that occurs as a result of the struggle with highly challenging life crises, describing the experience of individuals who do not only recover from trauma but also discover it as an opportunity for further individual development. Those individuals overcome trauma with improved psychological functioning in specific domains (7). PTG has been reported by a significant number of people who have encountered major life challenges, resulting in such factors described as new possibilities, relating to others, personal strength, spiritual change, and appreciation of life (8).

Concerning the negative and positive change of the struggle with life-threatening events, the relation between PTSD and PTG remains controversial. It is natural to hold the point of view that PTSD correlates negatively with PTG. However, some studies showed that there is a positive correlation between them. Kleim and Ehlers reported that there is a significant curvilinear association between PTSD and PTG. Higher PTG levels were associated with greater PTSD and depression symptom severity (9). Solomon and Dekel declared that both linear and quadratic associations were found and that prisoners of war exhibited higher levels of PTSD and PTG (10). Similarly, a strong positive correlation was also observed between PTSD and PTG among undergraduate students (11). Moreover, Chang et al. found a positive association between PTG and PTSD through a sample of 2,300 earthquake survivors 1 year after the 2008 Wenchuan earthquake (12). The findings indicate that these two trauma manifestations could coexist and are not mutually exclusive. However, some researchers have found a weak negative correlation between PTSD and PTG, for example, among Israeli youth exposed to terror incidents and Chinese survivors after an earthquake (3, 13). On the contrary, others have reported a negative correlation between PTG and psychological distress or probable PTSD (14).

Traumatic events can cause a series of strong emotional responses, such as fear, anxiety, and depression. Many studies support the idea that emotional function plays a very important role in trauma and post-traumatic experience. To a large degree, the characteristic symptoms of PTSD, namely intrusion, anxiety, arousal, and “flashbacks” of the traumatic event, suggest abnormalities in the processing of emotion, such as emotion associated with traumatic, emotional material. Evidences from both animal and human studies support the opinion that the dysfunction of emotional functioning is an important factor leading to the development and maintenance of PTSD (15, 16). Ehring and Quack used questionnaires to assess characteristics of emotion regulation, showing that PTSD symptom severity was significantly associated with all variables assessing emotion regulation difficulties (17). A study from single photon emission computerized tomography investigated the brain mechanism of the emotional dysfunction. The results indicated that, compared with the other subjects, activation in the region of the left amygdala/nucleus accumbens was found in PTSD patients only, implicating regions of the “limbic” brain, which may mediate the response to aversive stimuli in healthy individuals and in patients suffering from PTSD (18).

Compared with the positive changes followed by trauma, such as PTG, research on the negative sequelaes of trauma, especially PTSD, has grown rapidly during the last decades. Furthermore, rapid progress in our understanding of negative emotions and psychopathological conditions has been made due to the study of neural and other biological correlates of these phenomena. Only in recent years, the positive changes following trauma have been studied systematically.

Based on the reviews above, our study aims at: (1) investigating the relationship between PTSD and PTG, trying to find the correlation between them; (2) exploring the trauma-related factors that influence e PTSD and PTG; (3) comparing the emotional response performance of PTSD and PTG. We hope that this study may provide a new perspective and become an important supplement for the current trauma-related research effort.

## Materials and Methods

### Participant

On Wednesday, 12 August 2015, a series of explosions that killed 165 people and injured hundreds of others occurred at a container storage station at the Port of Tianjin. First, for the purpose of assessing the relationship between PTSD and PTG, and exploring the trauma-related factors that influence PTSD and PTG, a sample was collected from the companies in the surrounding areas of the explosive center in Tianjin port, and most dormitories of the participants were destroyed by the explosions. Every participant was in the explosive region and experienced the explosions when it happened. In all, 107 people were excluded because of uncompleted questionnaire items or lack of cooperation, and 76 people were excluded because of the blurred writing, so 2,395 participants were included in this sample.

Second, in order to compare the emotional response performance of PTSD and PTG, a sample of 90 participants who scored highly for PCL-C or PTGI was selected from the above sample, and the 90 participants was divided into three groups: PTSD, PTG, and control group.

The instructions were clearly explained to all subjects prior to answering the questionnaires and doing the emotional response task, making sure that all participants fully understand and consent for voluntary participation in this research study. The basic information of the samples is presented in Table 1.

**Table 1:** **Basic information of participants**.

		Sample 1 (*n* = 2,395)		Sample 2 (*n* = 90)	
		*N*	%	*N*	%
Gender	Male	1,327	55.41	48	53.33
	Female	1,068	44.59	42	46.67
Age (M ± SD)	28.43 ± 6.46			22.94 ± 6.07	
Age group	16–25	652	27.22	25	27.78
	26–35	446	18.62	23	25.56
	36–45	815	34.03	26	28.89
	≥45	482	20.13	16	17.78
Witnessed explosions	Yes	291	12.15	18	20.00
	No	2,104	87.85	72	80.00
Witnessed corpse	Yes	304	12.69	22	24.44
	No	2,091	87.31	68	75.56
Distance from center	≤3 km	265	11.06	27	30.00
	≥3 km	2,130	88.94	63	70.00
Loss	None	1,322	55.2	16	17.78
	A little	838	34.99	32	35.56
	Much	145	6.05	25	27.78
	A lot	90	3.76	17	18.89

### Ethics Statement

This study was approved by the ethics committee of the Institute of Psychology, Chinese Academy of Sciences. All methods and protocals in the experiment were performed in accordance with the relevant guidelines and regulations of the approved methods and protocols. The procedure of the study was fully explained to the participants, and informed written consent was obtained from each participant before the study.

### Measures

All participants were recruited and asked to complete a set of questionnaires, including the basic information, PTSD Checklist-Civilian Version (PCL-C), Post-traumatic Growth Inventory (PTGI). Details of the measures will be further introduced as follows.

PCL-C consists of 17 items which correspond directly to DSM-IV PTSD symptoms. Each item is rated on a 5-point Likert scale using anchors ranging from one “not at all” to five “extremely” (19). The Chinese version of the PCL was adapted by a stringent two-stage process of translation and back translation (20). In this study, Cronbach’s α for the scale was 0.931 and participants of the sample were instructed, respectively, to complete the PCL-C referring to the “explosions of Tianjin.”

Post-traumatic Growth Inventory consists of five subscales comprising 21 items: personal strength, new possibilities, relating to others, appreciation of life, and spiritual change (8). The Chinese version of PTGI was developed through translation and back translation. Items are rated on a 6-point Likert scale, ranging from 0 to 5. The internal consistency of mean PTGI scores was very good in the sample (21 items, α = 0.942).

The degree of trauma exposure was measured with the (a) direct exposure (four items: the distance from the explosion center, if they witnessed explosions, if they witnessed a corpse, if they witnessed death); (b) close ones’ exposure to the explosion-related stressors (three items: if they had any family members, relatives, friends, or co-workers wounded or killed during or after the explosions); (c) loss (two items: the impact of the explosions on their house and property).

### Materials

In order to examine emotional response effects, we administered affective priming tasks developed by Fazio et al. (21). Prime pictures (16 positive, 16 negative) and target pictures (16 positive, 16 negative) were selected on the basis of a preliminary rating study in which the participants (*N* = 90) had to judge the affective level of the 64 colorful pictures on a 9-point rating scale ranging from −4 (very negative) to +4 (very positive). All pictures originated from the International Affective Picture System (IAPS, Centre for the Psychophysiological Study of Emotion and Attention, 1994). Positive and negative targets differed significantly on the affective dimension. The prime picture, mask picture, and target picture were presented against the black background of a (30 cm × 38 cm) computer monitor *via* the E-Prime software (E-Prime 2.0, Psychology Software Tools, Inc., Pittsburgh, PA, USA). The presentation order of slides was randomized among subjects.

### Method

First, the questionnaires include basic information, PCL-C, PTGI, and trauma exposure level survey were completed at the participants’ companies with the guidance of investigators, so that when they had any question, they could receive immediate help. The data collection was conducted approximately 3 months after the serious explosions and lasted for 2 weeks.

Second, the 90 participants were tested individually in a dimly lit and quiet room. Practice and experimental trials were informed to the participants through the instructions on the computer screen. At the beginning of the procedure, the fixation was presented for 100 ms, after it disappeared, a prime picture was immediately presented for 200 ms at the same position, then the mask picture was presented for 100 ms at the same position. When the mask picture vanished, the target picture was finally presented at the same position. The participants were asked to judge whether the target picture was perceived positive or negative by pressing the key “F” for positive and “J” for negative. During the response phase of the experiment, the participants were instructed to press the correct key as fast as possible. In order to avoid the participants’ anticipation, the attribute of the pictures presented in a random order. The whole experiment consisted of 128 trials, subdivided into four blocks (negative–positive, negative–negative, positive–negative, positive–positive) of 32 trials in which each prime picture was presented once, together with each target picture, each picture was thus presented four times. E-Prime 2.0 (2.0.10.200) was used to control all experiments.

### Data Analysis

The analysis of this study proceeded as follows. First, all data were imported from E-Prime 2.0.txt files into SPSS. Second, the descriptive statistics and Pearson’s correlation analysis for the scales were conducted through the Statistical Package for the Social Sciences (SPSS version 20.0). Third, group (PTG, PTSD, and control) × prime (positive, negative) analysis of variance (ANOVA) was conducted for the response time data.

## Results

Overall, PCL-C scores averaged 29.69, with SD = 7.89. PTG scores with a mean score of M = 54.09, SD = 20.18. The details of the measures are presented in Table 2.

**Table 2:** **Descriptive statistics of measures**.

Variable		M ± SD
PCL-C	Intrusion	8.90 ± 3.58
	Anxiety	3.49 ± 1.62
	Numbing	8.02 ± 3.24
	Dysphoric arousal	5.57 ± 2.36
	Anxious arousal	3.86 ± 1.84
	Total	29.69 ± 7.89
Post-traumatic Growth Inventory	Relationship	19.33 ± 7.61
	New possibilities	11.14 ± 5.36
	Personal strength	11.80 ± 4.67
	Spiritual change	3.63 ± 2.72
	Appreciate life	8.08 ± 3.05
	Total	54.09 ± 20.18
Direct explosion		4.35 ± 0.84
Explosion-related stressors		4.99 ± 2.28
Loss		2.79 ± 1.09

The Pearson bivariate correlation was conducted to investigate the correlation between PTSD and PTG. Although the results indicated that PCL-C negatively correlated with PTGI (*r* = −0.05, *p* = 0.04), the correlation coefficient was generally weak. Moreover, PCL-C was strongly negatively associated with relationship (*r* = −0.09, *p* < 0.001), new possibilities (*r* = −0.06, *p* = 0.004), and personal strength (*r* = −0.10, *p* < 0.001) items, but positively associated with spiritual change (*r* = 0.06, *p* = 0.002) and appreciation of life (*r* = −0.07, *p* = 0.001). Meanwhile, PTGI was negatively associated with the two cluster of PTSD, numbing (*r* = −0.12, *p* < 0.001) and dysphoric arousal (*r* = −0.08, *p* < 0.001), but not associated with the other three clusters. Results of the Pearson correlations are presented in Table 3.

**Table 3:** **The correlations of post-traumatic stress disorder Checklist-Civilian Version (PCL-C) and Post-traumatic Growth Inventory (PTGI)**.

Variables	1	2	3	4	5	6	7	8	9	10	11
1. PCL-C	–										
2. Intrusion	0.89**	–									
3. Anxiety	0.79**	0.71**	–								
4. Numbing	0.86**	0.65**	0.62**	–							
5. Dysphoric arousal	0.84**	0.65**	0.55**	0.67**	–						
6. Anxious arousal	0.79**	0.64**	0.53**	0.57**	0.67**	–					
7. PTGI	−0.05*	0.02	0.02	−0.12**	−0.08**	0.01	–				
8. Relationship	−0.09**	−0.02	−0.03	−0.17**	−0.11**	−0.03	0.94**	–			
9. New possibilities	−0.06**	0	0.02	−0.09**	−0.11**	−0.02	0.90**	0.77**	–		
10. Personal strength	−0.10**	−0.05*	−0.04*	−0.15**	−0.11**	−0.04*	0.89**	0.79**	0.75**	–	
11. Spiritual change	0.06**	0.07**	0.09**	0.03	0.01	0.08**	0.67**	0.54**	0.61**	0.49**	–
12. Appreciation of life	0.07**	0.12**	0.08**	−0.03	0.05**	0.10**	0.76**	0.67**	0.59**	0.64**	0.41**

*p < 0.05.

**p < 0.01.

Another Pearson bivariate correlation was conducted to evaluate the factors of the explosions, such as the direct exposure, close ones’ exposure, and the loss. Results showed that PCL-C was positively correlated with the direct exposure (*r* = 0.24, *p* < 0.001), close ones’ exposure (*r* = 0.65, *p* < 0.001), and the loss (*r* = 0.29, *p* < 0.001). On the contrary, the correlation coefficients of PTGI and the three factors were generally weak. The details are presented in Table 4.

**Table 4:** **The correlations of post-traumatic stress disorder Checklist-Civilian Version (PCL-C), Post-traumatic Growth Inventory (PTGI), and trauma exposure**.

Variables	1	2	3	4
1. PCL-C	–			
2. PTGI	−0.05*	–		
3. Direct exposure	0.24**	0.02	–	
4. Explosion-related stressors	0.65**	−0.09**	0.14**	–
5. Loss	0.29**	0.07**	0.63**	0.18**

*p < 0.05.

**p < 0.01.

Because PCL-C demonstrated a very weak correlation with PTGI, we selected a sample of 90 participants and divided them into three groups: (1) the control group (*n* = 30), scores of PCL-C and PTGI are both weak relatively, mean_PCL-C_ = 26.93, SD_PCL-C_ = 3.81, mean_PTGI_ = 49.00, SD_PTGI_ = 4.27; (2) the PTSD group (*n* = 30), the participants scored high in PCL-C but low in PTGI, mean_PCL-C_ = 52.53, SD_PCL-C_ = 2.81, mean_PTGI_ = 50.80, SD_PTGI_ = 3.87; (3) the PTG group (*n* = 30), compared with the PTSD group, the PCL-C’s scores are low but the PTGI’s scores are high, mean_PCL-C_ = 30.60, SD_PCL-C_ = 3.70, mean_PTGI_ = 86.53, SD_PTGI_ = 3.23. The details of the group are presented in Table 5. The one-way ANOVA analysis revealed that the PCL-C scores of the three groups are different [*F*(2,89) = 183.008, *p* < 0.001], and the LSD test showed that the PCL-C in the PTSD group is higher than in the control (*p* < 0.001) and PTG group (*p* < 0.001), and that there is no difference between the control and PTG group (*p* = 0.270). Meanwhile, the one-way ANOVA analysis also showed that the PTGI scores are different among the three groups [*F*(2,89) = 923.704, *p* < 0.001], and the LSD test showed that the PTGI in the PTG group is higher than in the control (*p* < 0.001) and PTSD group (*p* < 0.001), and that there is no difference between the control and PTSD group (*p* = 0.270).

**Table 5:** **The mean scores and SD of PCL-C and Post-traumatic Growth Inventory (PTGI) among the control, post-traumatic stress disorder (PTSD), and PTG group**.

Group	Survey	Mean	SD
CON (*n* = 30)	PCL-C	26.93	3.81
	PTGI	49.00	4.27
PTSD (*n* = 30)	PCL-C	52.53	2.81
	PTGI	50.80	3.87
PTG (*n* = 30)	PCL-C	30.60	3.70
	PTGI	86.53	3.23

**Figure 1:**
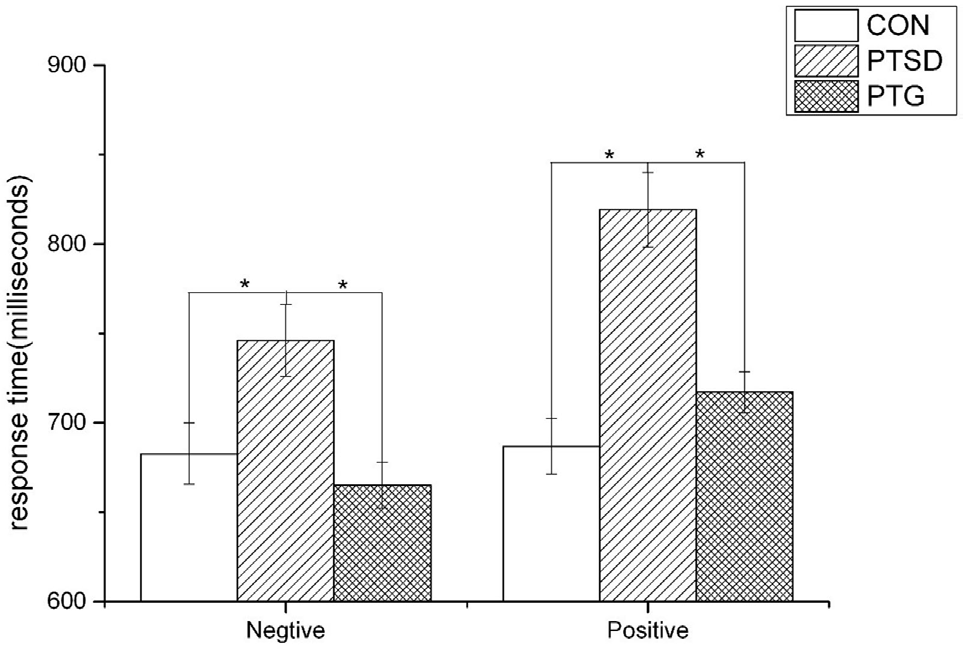
The response time of negative and positive priming among three groups (*p < 0.001).

A mixed design, repeated measures ANOVA for response time revealed no significant overall effect of priming [*F*(1,87) = 0.255; *p* = 0.615], and no significant effect of (priming × group) interaction [*F*(2,87) = 2.614; *p* = 0.079]. However, a significant effect of group [*F*(2,87) = 16.146; *p* < 0.0001] was found. LSD test revealed that response time of PTSD group is longer than control (*p* < 0.0001) and PTG group (*p* < 0.0001), but there is no difference between the control and PTG group (*p* = 0.120) (Figure 1).

## Discussion

Several studies focused on the relationship between PTG and PTSD and reached different conclusions, some held the point of view that there was a positive correlation, while others suggested that there was a negative correlation between them. Based on a sample of 2,395 participants, the results of this research revealed that 16.9% of the participants suffered from moderate to very severe PTSD symptoms 3 months after the explosions, suggesting that PTSD was a serious problem among the people in the adjacent areas of the explosions. Meanwhile, the prevalence of PTG among the participants was 8.4%, which was relatively low compared with PTSD. The correlation between PTSD and PTG was very weak, with a correlation coefficient of −0.05, they are not correlated to a certain degree. Moreover, the correlations of the clusters of PTG and PTSD were weak too. We explored the reasons for the controversial relationship between PTSD and PTG. The level of trauma exposure could be an important reason of influence, and a low level could weaken the correlation between PTSD and PTG. The exposure level of the participants in this study was generally low.

The results also suggest that among participants with high PTSD intensity, most suffered strong traumatic stress, such as direct exposure, close one’s exposure, and loss. On the contrary, PTG was not strongly correlated with the above factors. Considering the perspective that PTG may represent a process, style, and outcome of coping and struggle with adversities after a traumatic event (22), the personality traits may play a more important role in PTG compared with the trauma. Our previous research demonstrated that PTG was positively associated with resilience and reflective rumination (13).

The characteristics of emotional response of PTSD and PTG were investigated at the same time in this study. The results indicated that, compared with the control and PTG subjects, in the condition of both positive and negative affective priming, PTSD subjects needed to invest more time to perform affective picture recognition and judgment. The experience of trauma produces very serious emotional problems, such as emotional numbness. PTSD is associated with the disorder of emotional process and reaction. Many trauma survivors also report restrictions in their emotional experience – a phenomenon most commonly referred to as emotional numbing (23). The changes of PTSD criteria in DSM-5 have attracted much concern. DSM-5 pays more attention to emotional problems and proposes a new cluster of negative cognitions and mood focused on the negative trauma-related emotions (6).

As for the response time of PTG, contrary to our expectation, the data showed that there was no difference between the PTG group and the control group. According to previous studies, the positive changes of PTG include those associated with emotions or cognitions. However, we noticed that almost all studies used the surveys to investigate the changes of emotion and cognition for PTG, and the changes were depended on the subjects self-reporting or self-experiencing to a certain degree. Perhaps one might also interpret this phenomenon as a form of secondary gain, deserving further cross-disciplinary exploration. Although our results showed that PTG manifested no change on emotional function through the affective priming paradigm, this was the first study to our knowledge, which focused on the emotional changes for PTG from the behavioral perspective.

There are some limitations to our study. First, we know that PTSD and PTG may be two different dynamic processes of post-traumatic experience, they may interact with each other (24, 25), whereas, this research is a cross-sectional study, and the data collection was conducted approximately 3 months after the serious explosions. So to a certain degree, the conclusion of the relationship between PTSD and PTG we have drawn from the data is isolated and incomplete. To overcome this shortage, we will continue to collect the data at the time of 6, 12, and 24 months after the explosions, investigating the dynamic correlation between PTSD and PTG, making this research to be a retrospect and longitudinal work. Second, because PTSD was not correlated with PTG in study 1, we put them into an opposite position in the emotional response study, divided participants into the groups of PTSD, PTG, and control, and took PTSD and PTG are two different and opposite terms for granted. However, some researches hold the view that PTSD and PTG were coexisted and interacted (26, 27). To solve this problem, we need to develop a new method of group dividing in the future study. Third, some other factors should be considered when we investigate the correlation of PTSD and PTG, such as personality trait and social support (28, 29).

## Author Contributions

CW designed the experiment, analyzed the data, and wrote the main manuscript text, JH prepared the materials and equipments and did a lot work for the data collection and analysis, YZ made a lot guidance and all funds supports for the whole research, ZL prepared the participants and room conditions, WH revised the manuscript. All authors reviewed the manuscript.

## Conflict of Interest Statement

The authors declare that the research was conducted in the absence of any commercial or financial relationships that could be construed as a potential conflict of interest.
